# Nutritional aspects in autoimmune diseases undergoing hematopoietic stem cell transplantation: overview and recommendations on behalf of the EBMT ADWP and Nurses Group

**DOI:** 10.3389/fnut.2024.1394518

**Published:** 2024-05-09

**Authors:** Chiara Gandossi, Helen Jessop, Anne Hahn, Lisa Heininger, Jörg Henes, Alexia Marina Radaelli, Anna Carmagnola, Enrico Morello, Chiara Renica, Alice Bertulli, Lorenzo Lazzari, Michelle Kenyon, Tobias Alexander, Ariadna Domenech, Raffaella Greco

**Affiliations:** ^1^Hematology and Bone Marrow Transplantation Unit, IRCCS San Raffaele Hospital, Vita-Salute San Raffaele University, Milan, Italy; ^2^Department of Haematology, Sheffield Teaching Hospitals NHS Foundation Trust, Sheffield, United Kingdom; ^3^Department of Internal Medicine II (Hematology, Oncology, Clinical Immunology and Rheumatology), University Hospital Tuebingen, Tuebingen, Germany; ^4^Blood Diseases and Cell Therapies Unit, Bone Marrow Transplant Unit" ASST-Spedali Civili" Hospital of Brescia, Brescia, Italy; ^5^Department of Clinical and Experimental Sciences, University of Brescia, Brescia, Italy; ^6^Department of Haematology, King's College Hospital, London, United Kingdom; ^7^Department of Rheumatology and Clinical Immunology, Charité - Universitätsmedizin Berlin, Corporate Member of Freie Universität Berlin, Humboldt-Universität zu Berlin, Berlin Institute of Health, Berlin, Germany; ^8^Bone Marrow Transplant Unit, Department of Hematology, Hospital Clínic of Barcelona, Barcelona, Spain

**Keywords:** nutrition, nutritional support, hematopoietic stem cell transplantation, autoimmune diseases, multiple sclerosis, systemic sclerosis, Crohn’s disease

## Abstract

Autoimmune diseases (ADs) represent a heterogeneous group of conditions affecting 5–10% of the global population. In recent decades, hematopoietic stem cell transplant (HSCT), mainly autologous, has been successfully adopted to treat patients affected by severe/refractory ADs. In this context malnutrition has a detrimental impact on relapse, mortality, infection rate, engraftment, long-term survival, and prolongation of hospitalization. However, in this population, the management of nutrition should be improved since nutritional assessment is partially performed in routine clinical practice. A panel of nurses and physicians from the European Society for Blood and Marrow Transplantation (EBMT) reviewed all available evidence based on current literature and expert practices from centers with extensive experience in HSCT for ADs, on the nutritional management of ADs patients during HSCT procedure. In this context, adequate nutritional status predicts a better response to treatment and improves quality of life. Herein, a systematic and comprehensive monitoring of nutritional status before, during and after HSCT, with adequate nutritional support in the case of ADs patients, in addition to assessing the dietary requirements associated with HSCT has been covered. Moreover, given the singularity of each AD, the underlying disease should be considered for an appropriate approach. The management and evaluation of nutritional status must be carried out by a multidisciplinary team to assess the needs, monitor the effectiveness of each intervention, and prevent complications, especially in complex situations as patients affected by ADs.

## Introduction

Autoimmune diseases (ADs) have had an increasing incidence in recent years (1). They constitute a heavy burden for affected patients, also resulting in considerable socioeconomic costs (2).

**Figure 1:**
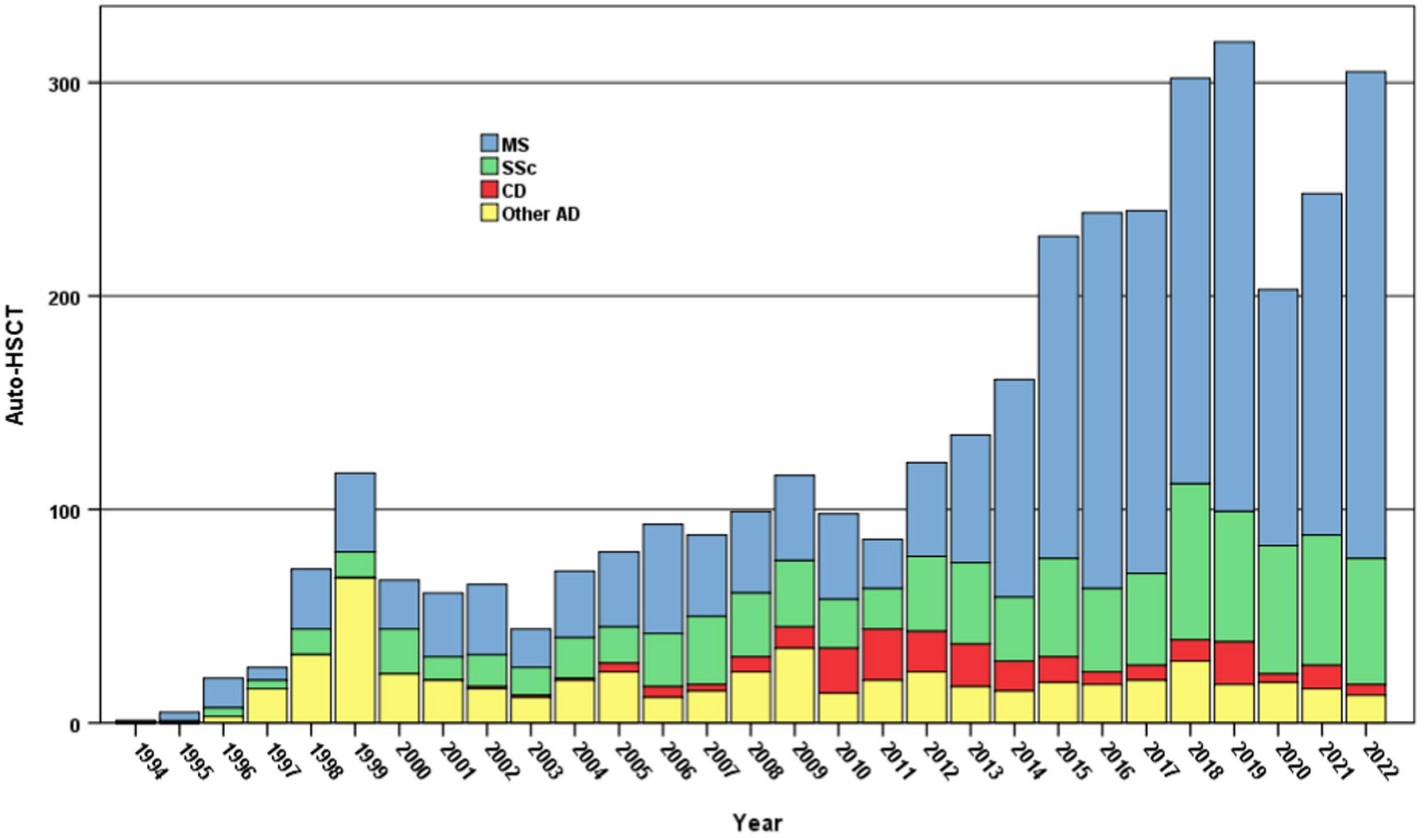
Autologous HSCT for ADs in adult patients - 1994-2022. Number of autologous HSCT reported to the EBMT-ADWP Registry data, per year and indication, from 1994 through 2022 (*n* = 3,831). AD, autoimmune disease; ADWP, Autoimmune Diseases Working Party; EBMT, European Society for Blood and Marrow Transplantation; HSCT, hematopoietic stem cell transplantation; MS, multiple sclerosis; SSc, systemic sclerosis; CD, crohn’s disease.

Over the last 3 decades, hematopoietic stem cell transplantation (HSCT) has been increasingly considered as treatment option for patients affected by severe/refractory ADs, inducing a total and stable remission of the disease (3–5). The European Society for Blood and Marrow Transplantation (EBMT) Autoimmune Diseases Working Party (ADWP) has played a central role in this development, with overall 3,992 HSCT (3,831 autografts and 91 allografts) registrations for ADs in adult patients (median age 39 years, range 18–76). Recent data has improved the evidence to support HSCT in Multiple Sclerosis (MS) and Systemic Sclerosis (SS), along with a wide range of other indications (3). More than half of the HSCTs registered in the ADWP database (Figure 1) have been performed on patients diagnosed with MS, followed by SS, and in a smaller group with Crohn’s Disease (CD).

The continuous effort in ameliorating supportive care represents one of the cornerstones to improve the overall HSCT outcome in these patients. Nowadays, more than ever, nutritional support is considered a key feature for the success of HSCT procedure.

The conditioning regimen administered before HSCT generally causes inflammatory damage to the gastrointestinal tract, which determines the disruption of the mucosal barrier, leading to mucositis, loss of appetite, diarrhea, nausea, vomiting and dysgeusia (6). These symptoms, together with the catabolic state and infections sequelae, often result in suboptimal nutritional intake followed by loss of weight and lean body mass before and after transplant (7). These gastrointestinal complications, added to psycho-emotional factors, fatigue, decreased physical activity and prolonged hospitalization, are the main reasons for a severe and rapid nutritional decline after transplant (8, 9). The nutritional status deeply impacts on hospitalization, quality of life, infection rate and survival after HSCT (10–12). Moreover, in the allogeneic setting, nutritional impairment and gastroenteric damage may also trigger intestinal graft-versus-host disease (GvHD), which can further negatively affect food intake and gut absorption (6). It is crucial to consider that these alterations tend to persist long after transplant, having reported that 50% of the patients have not regained their weight after a year from HSCT, a fact that makes even more relevant the need to monitor the nutritional status of our patients before, during and after the procedure (13). In this context, the intestinal microbiota deeply affects the clinical outcome of HSCT patients in terms of mortality (14), infections (15, 16), and GvHD (17) including severely treatment-resistant AD patients, because of its interaction with the host’s immune system and its contribution toward immune-related complications related to the nature of the disease and its undergoing therapy (3). Moreover, the loss of fecal intestinal diversity in the peri-engraftment period after autologous HSCT is associated with decreased risk of death or progression (18).

Even if all these implications are widely studied, in real-world clinical practice, both awareness and consideration of nutritional issues remain poor and nutritional assessment is not routinely performed (6).

The aim of this review, developed on behalf of the European Society for Blood and Marrow Transplantation (EBMT) Autoimmune Diseases Working Party (ADWP) and Nurses Group, is to summarize the current literature and expert practices from experienced centers about nutritional assessment in AD patients undergoing HSCT, developing practical expert-based recommendations for the day-to-day care by healthcare professionals.

## Nutritional screening and support during HSCT procedure

In all patients eligible for HSCT, the clinical guidelines of the European Society for Parenteral and Enteral Nutrition (ESPEN) recommend the systematic and comprehensive monitoring of nutritional status before, during and after transplant, together with an adequate nutritional support, when required (19, 20). Literature shows that between 10 and 15% of patients have malnutrition prior to transplant (6).

For this reason, before HSCT admission, all patients must receive a nutritional assessment and screening in order to implement corrective measures as soon as possible (6). There are different tools to assess the nutritional status of the patients and detect the risk of malnutrition, but currently one of the most used is the Malnutrition Universal Screening Tool (MUST). This tool helps in detecting not only patients with malnutrition but also those at risk and who could benefit from preventive nutritional interventions (21).

Among nutritional assessment tools, Patient-generated Subjective Global Assessment (PG-SGA) has the advantage of scoring patients’ reported outcomes regarding gastrointestinal symptoms, food intake and subjective perception. The alpha-numeric score could be useful in the assessment of malnutrition, triage and monitoring during the follow-up (22).

Patients at nutritional risk should undergo a complete evaluation to effectively diagnose malnutrition, through the assessment of phenotypic and etiologic criteria. Body composition analysis could help physicians in detecting malnourished patients who may have low muscle mass or sarcopenia, which are often hidden and overlooked, especially in patients with normal weight or excess adiposity (6). When malnutrition is diagnosed, a nutritional intervention aimed at maintaining or recovering appropriate nutritional status should be promptly initiated and tailored according to current nutritional status, clinical condition, planned treatment, and the expected outcome (6).

Where an assessment has not been carried out before, a screening for determining the risk of malnutrition should be performed using validated tools within 48 h of hospital admission, according to the Global Leadership Initiative on Malnutrition (23).

During the post-transplant period, patients may present increased nutritional requirements combined with impaired metabolism and difficulty in maintaining oral intake (19). Some suggested interventions during the procedure should involve adjusting the intake, modifying the diet and evaluating the need to administer oral nutritional supplements (ONS). In patients predicted to be unable to ingest and absorb adequate nutrients for a prolonged period, the implementation of Parenteral Nutrition (PN) or Enteral Nutrition (EN) is indicated (19). ESPEN guidelines prefer EN as first choice for nutritional support, associated with a lower incidence of infective complications, better overall survival and faster neutrophil engraftment. In the context of allogeneic HSCT, EN reduces the incidence of acute GvHD, mainly for severe and gut forms (24). In pediatric population undergoing HSCT, EN promotes a more favorable intestinal microbial network as compared to PN (25). Moreover, it is more physiological, and contributes to the preservation of gut mucosal integrity and function. On the other hand, limitations in the choice of EN, together with difficulties in positioning and tolerating the nasogastric tube or stoma, usually lead to a preferential use of PN in patients undergoing HSCT (6).

## Nutrition and oropharyngeal mucositis

Oropharyngeal Mucositis (OM) refers to the inflammation of the oral and gastrointestinal mucosa. It is characterised by erythema and ulceration of the mucosal membrane which may result in pain, difficulty in swallowing, impaired taste and quality of life (QoL) for several weeks (26, 27). OM is common in patients receiving myeloablative doses of chemotherapy and can affect around 60 to 80% of HSCT recipients (28).

In this regard, a recent single centre, retrospective study demonstrated that patients receiving a myeloablative conditioning experienced grade 2–4 and grade 3–4 mucositis in 61 and 41% of cases, respectively (29). In this study, the mean duration was 4.2 days for grade 2–4 and 1.4 days for grade 3–4. In intensive regimes, based on busulfan or total body irradiation, OM may be prolonged.

OM may be prevented by cryotherapy, expecially in High Dose Melphalan conditioning (30).

OM may be reduced by good oral care, which is based on four key principles: accurate assessment, individualized care plans, initiation of timely preventative measures and correct treatment (31). Assessment should begin prior to commencing treatment and include a dental evaluation and an accurate patient history to assess risk factors for oral damage (32).

Inspection of the oral cavity should include the cheeks, lips, soft palate, floor of the mouth and tongue and should be initiated on admission, continued daily throughout treatment and at appropriate time points post discharge, until full healing has occurred. Patient education is a key aspect and patients should receive both verbal and written information about oral care to facilitate prompt reporting of problems.

As OM develops, healthcare professionals should manage pain medication and nutritional support (4). Although these measures may not reduce the severity of OM, many products can provide patient comfort and therefore help to maintain some food and fluid intake and enable rest (32). Adequate nutrition is vital to maintain mucosal integrity and reduce deterioration of existing damage enhancing repair (32).

Mucositis also affects the lower gastrointestinal tract and may lead to profuse diarrhea. Patients with CD, for example, will have a higher predisposition to this event. Other causes, such as infections, should be determined by stool cultures and appropriate treatment should be instigated. If cultures are negative, an antidiarrheal agent can be safely prescribed and close monitoring of fluids and electrolytes will be required, including liquid replacement as necessary (32).

In summary, the severity of OM in patients undergoing HSCT for ADs is very variable and centers will need to develop strategies to manage it depending on the underlying disease of the patient and the conditioning regimen used.

## Nutrition and infections

Despite advances in supportive care, infections remain a significant cause of morbidity and mortality in the setting of HSCT (33, 34). The resultant injury on the gastrointestinal epithelial barrier by chemotherapy (CT) or radiotherapy (RT), and the extensive use of antibiotics during the neutropenic phase, may lead to gut microbiota dysbiosis (35). When dysbiosis occurs within the damaged gut epithelium and the equilibrium of the microbial composition becomes fluctuant, the dominant bacteria can potentially invade the bloodstream, causing bacteremia (16). These considerations make the intestinal microbiota an independent predictor of clinical outcome and mortality in patients undergoing HSCT (14, 15, 36). Diet and nutrition could positively affect microbiome diversity during HSCT procedure, as growing evidence suggests a link between them (37).

Various measures have been adopted to minimize the incidence of infections, including the use of a low-bacterial diet (LBD) that excludes raw food, fresh fruits, and vegetables (33). The rationale behind the use of LBD is to prevent the introduction of dangerous bacteria into the gastrointestinal tract, already damaged by CT or RT (33, 38). Regarding nutritional support with LBD or non-restrictive diet (NRD), the literature points out that the former has shown no benefit on infection rates or survival, in contrast with the routine use of low-microbial foods in clinical practice (39). Moreover, results from a recent randomized study by Stella et al. (33) demonstrated that the frequency of infections, deaths, nutritional outcomes, and acute GvHD was not different in patients receiving LBD versus NRD during the neutropenic phase after HSCT. These results suggest that the use of a restrictive diet is an unnecessary burden for patients’ QoL, even if, undoubtedly, data are more mature in the autologous HSCT setting in which LBD should no longer be recommended.

In the case of patients with ADs and an indication for transplant, in addition to assessing the nutritional requirements associated with the HSCT, it is essential to consider the underlying disease, since the singularity of each AD is important to carry out an adequate approach regarding nutritional indications.

## Specific nutritional aspects for multiple sclerosis

Multiple sclerosis (MS) is an autoimmune-mediated neurodegenerative disease of the central nervous system characterised by inflammatory demyelination with axonal transection (40). MS typically presents in young adults and can lead to physical disability, cognitive impairment, and decreased QoL (40). A total of 2.8 million people is estimated to live with MS worldwide (35.9 per 100,000 population). The mean age of diagnosis is 32 years, with a female to male ratio of 2:1 (41).

Autologous HSCT represent a standard-of-care approach in patients with highly active relapsing remitting MS failing to respond to disease modifying therapies (DMTs) (42). This population of patients need a formal metabolic-nutritional assessment, as some of the factors affecting MS pathogenesis are genetic and/or environmental and may include obesity and malnutrition (43). MS patients may experience signs and symptoms such as loss of vision, weakness, numbness, gait difficulty and bowel and bladder disturbances (9). In particular, the sensorimotor symptoms affecting the gastroenteric trait may lead to dysphagia and gastrointestinal problems such as fecal incontinence, diarrhea and constipation that have a strong impact on nutritional status (43).

Many studies have reported a significant connection between intestinal microbiota, eating habits, and the development of chronic-degenerative diseases such as MS (44). An increasing number of studies tried to investigate the potential role of the diet in MS (45).

Even though no definitive dietary recommendations have been scientifically proven to be beneficial in changing the course of MS, literature shows that maintaining balanced, low saturated fats and high-fiber diet, which is also rich in probiotics and vitamins, is associated with a decreased risk for MS-related disability and fatigue and with higher QoL (43). In facts, a low-fats diet reduces the risk of developing cardiovascular disease and diabetes, comorbidities associated with increased disability and decreased QoL in patients with MS (46).

It is also currently unclear whether salt, gluten or dairy are specifically harmful in MS, even though diets high in salt, processed gluten-free foods, and processed dairy substitutes are often less healthy and should be limited to benefit overall health (47–49). A diet high in vitamins (A, B2, B7, D), lipoic acid, omega-3 polyunsaturated fatty acids and amino acids (L-carnitine) may also have direct effects on the immune system and the brain (43). Moreover, various studies have encouraged the use of pre and probiotics in patients with MS due to their benefits in maintaining the homeostasis of the central nervous system and regulating the composition and balance of the gut microbiota (50). In the setting of allogeneic HSCT, there are several studies whose findings suggest that synbiotic intake before and during the conditioning regimen of HSCT patients may lead to a reduction in the incidence and severity of acute GvHD and contribute to the improvement of transplant outcomes, such as infections rate (51, 52). Indeed, more studies are needed to confirm the indication in prebiotics and probiotics intake in this specific population of patients undergoing HSCT. Moreover, the general deconditioning occurring after HSCT procedure can be associated with a transient increase in functional impairment and decrease in body mass, requiring an early implementation of rehabilitation strategies in MS population (53), together with nutritional support.

Rigorous head-to-head comparisons of dietary interventions are needed to address whether a specific diet is more effective at improving fatigue and QoL, as well as traditional MS outcomes such as relapse rate, MRI disease activity and accumulated disability.

## Specific nutritional aspects for systemic sclerosis

Systemic sclerosis (SSc) is a complex autoimmune connective tissue disease involving chronic and progressive tissue and organ fibrosis. Besides the skin, the gastrointestinal tract is the second most affected organ as approximately 70% of patients with SSc experience gastrointestinal symptoms within the first year (54, 55). Oropharyngeal and esophageal dysphagia, together with gastroesophageal reflux disease, malabsorption, constipation, diarrhea and fecal incontinence are the main complications (54). As the skin is the main organ involved, the lips are narrow and the forehead can no longer be moved; the opening of the mouth is restricted, the surface of the tongue becomes atrophic and its mobility restricted by the sclerotic hard lingual frenulum; moreover, the oral mucosa becomes dry (56). All the above aspects can lead to nutrient deficiencies because they affect food intake and absorption. The most frequently observed nutritional deficits in SSc are iron deficiency anemia and vitamin D deficiency (57).

Autologous HSCT is an effective therapeutic option for patients with severe SSc (58–60) as it can delay the progression of the disease and obtain improvements in organ function and skin condition (61). Autologous HSCT for early severe diffuse cutaneous SSc patients is now recommended as standard treatment (4). In this context, organ toxicity may be due to the preparative regimen and any underlying organ damage from SSc. The procedure comes with side effects affecting the gastrointestinal tract, and the prevalence of malnutrition is approximately 35% in SSc patients after HSCT (62). Gastrointestinal endoscopy has revealed gastric antral vascular ectasia (GAVE) in 22% of SSc patients screened before HSCT (63). Literature shows different ways to detect and document malnutrition as relying solely on BMI is not sufficient and the MUST tool is commonly used (64).

Nutritional therapy using enteral or parenteral support should be tailored to the clinical phenotype and severity of the disease. Parenteral nutrition is necessary when oral and/or enteral nutrition is inadequate or cannot be tolerated (65). As previously described, increased diarrhea may occur due to the SSc involvement of the gastrointestinal tract and may be related to food intolerances (particularly to gliadin, lactose and fructose) or can be the result of taking medication, particularly Nintedanib (66). Regarding the development of food intolerances, once identified through breath tests, the patient must receive nutritional advice on food modification. Anti-inflammatory diet and dietary measures aiming to promote gastric emptying, such as diets low in fats and fibers, are recommended (67). In addition, foods with a soft consistency are recommended. Eating several small portions throughout the day and drinking enough fluids is also helpful. An upright posture while eating and thorough chewing of food is recommended (54).

## Specific nutritional aspects for Crohn’s disease

Crohn’s Disease (CD) is a chronic, autoimmune, inflammatory bowel disease (IBD) that causes inflammation and irritation of the gastrointestinal tract, which evolves recurrently with flare-ups. The most frequent location is the terminal ileum and colon and the main symptoms presented in patients with CD include abdominal pain, diarrhea, weight loss, fever, fatigue and rectal bleeding (68–70). The global prevalence of IBD has been increasing since 2000, due to low mortality and improved survival among these patients, affecting 1 in 200 individuals in Western Countries (71). It usually occurs in people younger than 30 and the prognosis is associated with the time of evolution of the disease from diagnosis, the extension of the disease, the severity of flares, the types of complications, and the preceding history of surgical treatment. Treatment-refractory CD is associated with adverse QoL, recurrent hospitalization and treatment-related morbidity (72). In this scenario, autologous HSCT can be considered a therapeutic option in patients with objective evidence of inflammatory activity, severe course of the disease over the years, inadequate response to different therapies, and when surgery is not a viable option or is accompanied by significant risks (73). Many groups reported a correlation between chronic intestinal inflammation in IBD and intestinal microbiota characteristics (44).

In this specific setting, the nutritional status and the impact of conditioning regimens on the gastrointestinal system must be carefully considered and patients with CD should receive nutritional counseling before the start of the transplant procedure (74).

Malnutrition affects 65–75% of patients diagnosed with IBD due to reduced intestinal absorption, changes in the intestinal microbiota and varied symptoms, such as loss of appetite, nausea and vomiting. Gastrointestinal symptoms, such as nausea, vomiting, diarrhea, and abdominal pain, have been often reported in CD patients after HSCT. In addition, a series of nutritional deficiencies characterize this group of patients, such as a lack of folate and vitamins A and D, and B12 malabsorption in the case of extensive bowel resections (74). Nutrition plays a fundamental role in reducing the symptoms associated with CD or in maintaining remission (75), and those with refractory disease present frequently with signs of malnutrition and cachexia (73). Furthermore, TGF-beta2 enriched food for special medical purposes could reduce inflammation and bowel damage in active disease (76), incidence of severe and gut acute GvHD, pneumonia and hospitalization costs (77).

Artificial nutrition which may include EN or PN, is a part of the treatment, especially in those undergoing elective surgery. In fact, in some centers, parenteral nutrition and nil-by-mouth regimens are implemented from the day of stem cell infusion until the engraftment (19, 78). Meanwhile, literature indicates that during PN intestinal digestive enzyme activity and exocrine pancreatic function decrease and, in addition, there is evidence that the ileum and jejunum show reductions in mucosal mass and function. For this reason, it is fundamental to consider using a weaning protocol during the transition from PN to oral intake. Tolerance to oral intake should be confirmed and, during the transition, it is mandatory to avoid overfeeding and consider reductions if nutrition and hydration remain stable (79). The ESPEN guidelines recommend evaluating the need for oral or intravenous fluids and electrolyte supplementation, preparing a nutritional monitoring plan after PN is stopped to ensure a safe transition to complete oral nutrition.

Finally, when talking about oral diet, literature includes the use of other diets, such as the specific carbohydrate diet, the low FODMAP (fermentable oligosaccharides, disaccharides, monosaccharides, and polyols) diet and the semi-vegetarian diet (74).

## Recommendations for nurse management of nutritional status in ADs undergoing HSCT

Nurses play a fundamental role in the early detection of signs and symptoms of malnutrition and are pivotal in monitoring the effectiveness of supportive therapies. However, the literature lacks specific guidelines regarding nutritional support in AD patients undergoing HSCT.

**Figure 2:**
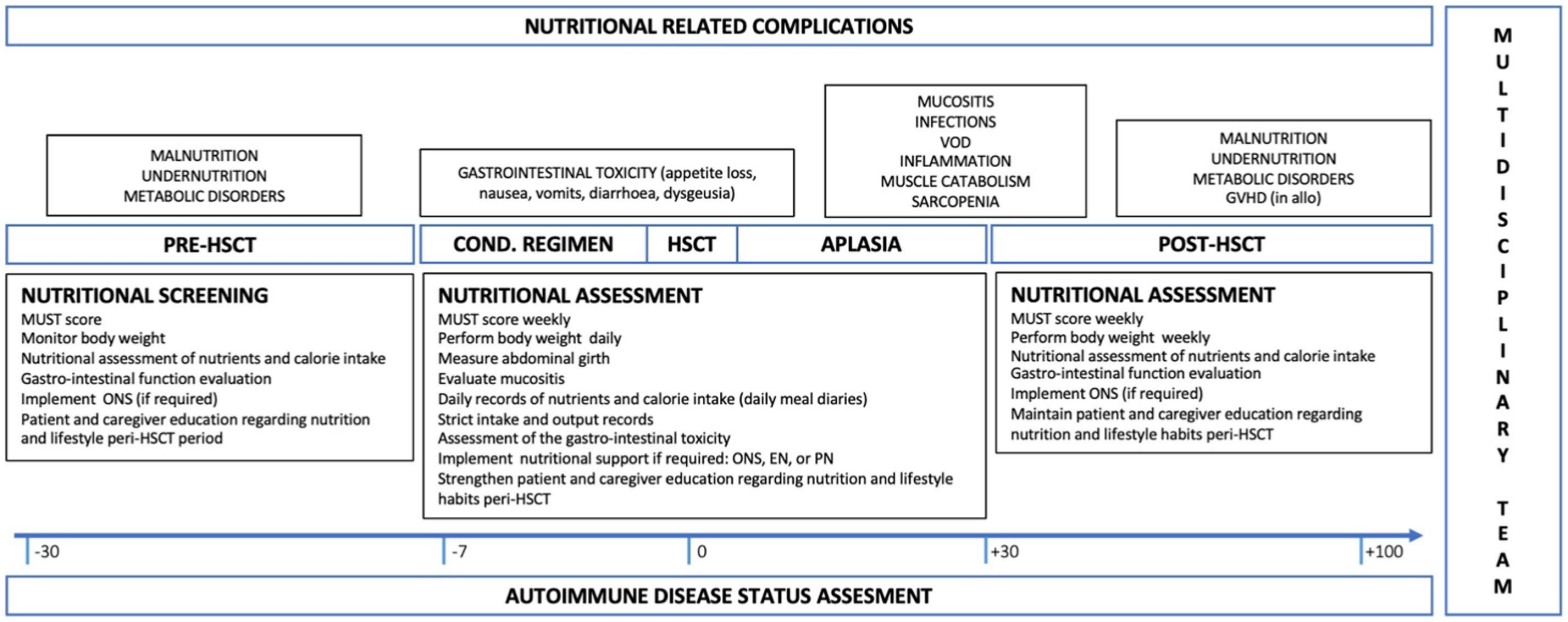
Flow chart of the ideal timing of assessment and interventions regarding nutritional support in AD patients undergoing HSCT. Nutritional interventions regarding each phase of the transplant procedure are here listed as regard to the literature suggestion and expert-base practices. All these interventions must be considered together with the nutritional-related complications, their severity and impact on patients’ food intake and QoL. AD, autoimmune disease; HSCT, hematopoietic stem cell transplantation; QoL, Quality of Life; MUST, Malnutrition Universal Screening Tool; ONS, Oral Nutritional Supplement; EN, Enteral Nutrition; PN, Parenteral Nutrition; VOD; Veno-Occlusive Disease; GvHD, Graft Versus Host Disease.

Based on current literature and expert practices from centers with extensive experience in HSCT for ADs, the panel of nurses and physicians from EBMT ADWP and Nurses Group has developed expert-based recommendations on nutritional management of AD patients undergoing HSCT (Figure 2). Guidance was primarily based on literature, collection of common practice data and personal opinions of the experts involved in the current manuscript. A significant preparatory work based on a comprehensive literature review was carried out by the experts, serving as the basis for the discussions. Given the lack of high-quality evidence in the context of ADs undergoing HSCT, recommendations were not graded. They therefore represent the consensus views of all the authors.

As nutritional support is a dynamic process across different phases, the first nutritional assessment must be performed before admission by a multidisciplinary team (8) or at least within 48 h after admission in the HSCT department (6). During the assessment, the healthcare team must evaluate the patient’s risk of developing malnutrition by obtaining and registering anthropometric parameters and baseline biochemical results together with using specific tools (i.e., MUST, NRS 2002, MNA, PG-SGA) (6). Other important information to collect is weight loss in the last 3–6 months and the patient’s inability to maintain his autonomy in daily living activities. If assessed before admission, as suggested by the centers involved, the collection of daily meal diaries helps to correct or integrate patients’ nutritional habits.

Once the assessment is done, the nursing team must carry out surveillance and control measures that facilitate the early detection of deterioration in the patient’s nutritional status since, in early stages, malnutrition can go unnoticed (6). It is mandatory to record complications associated with gastrointestinal toxicity derived from the conditioning regimen that may interfere with optimal oral intake and malabsorption. In the immediate post-HSCT, the team must consider the presence of neutropenia and the occurrence of infections that may influence patients’ nutritional status, producing weight loss and changes in body composition.

In addition, we recommend carrying out a nutritional record of daily intakes, to determine the appropriate inclusion of nutrients and calories, and the monitoring of MUST or similar tools once a week.

The nurse’s role is fundamental not only in detecting a possible deterioration in nutritional status but also in the education of patients and caregivers regarding what to eat, how to do that and when. In this regard, nurses must explain the rationale beside the nutritional support chosen and try to advocate patients’ preferences regarding nutrition when talking to the multidisciplinary team.

Importantly, nutritional support in patients undergoing autologous HSCT for ADs is a complex matter and must consider the underlying disease. Nutritional deficiencies and malabsorption must be suspected in the case of CD. Dysphagia and sensorimotor symptoms that affect the gastrointestinal tract are frequent in patients with MS, while chronic organ fibrosis that make chewing and swallowing difficult may occur in the case of SSc.

## Conclusion and future perspectives

The annual number of patients treated with autologous HSCT is constantly increasing (3) and AD are among the fastest growing disease category reported to the EBMT for autologous HSCT (80). An adequate nutritional status predicts a better response to therapy and improved QoL for patients undergoing HSCT procedure (6). For this reason, management and assessment of the nutritional aspects must be performed by a multidisciplinary team before, during and after transplant, to evaluate the nutritional needs, monitor the effectiveness of each intervention, and prevent complications. Education of patients and caregivers on these aspects is essential. Moreover, therapeutic approaches targeting microbiota, such as probiotics, prebiotics and postbiotics, can manipulate and influence the microbiota–host interactions (81–83).

In ADs, it is essential to consider the underlying disease as patients may present with different organs and systems affections, making it challenging to achieve adequate nutritional intake. There is evolving rationale, experience and forward vision of clinical experience by nurses and physicians in this setting. Further studies are needed to increase knowledge in this population, starting from the comparison of expert-based approaches and practices across the transplant and disease-specific communities.

## Author contributions

CG: Conceptualization, Data curation, Investigation, Methodology, Visualization, Writing – original draft, Writing – review & editing, Formal analysis, Validation. HJ: Writing – original draft, Writing – review & editing. AH: Writing – original draft, Writing – review & editing. LH: Writing – original draft, Writing – review & editing. JH: Writing – original draft, Writing – review & editing. AR: Writing – original draft, Writing – review & editing. AC: Writing – original draft, Writing – review & editing. EM: Writing – original draft, Writing – review & editing. CR: Writing – original draft, Writing – review & editing. AB: Writing – original draft, Writing – review & editing. LL: Writing – original draft, Writing – review & editing. MK: Writing – original draft, Writing – review & editing. TA: Writing – original draft, Writing – review & editing. AD: Data curation, Formal analysis, Investigation, Methodology, Supervision, Validation, Visualization, Writing – original draft, Writing – review & editing. RG: Conceptualization, Data curation, Formal analysis, Investigation, Methodology, Software, Supervision, Validation, Visualization, Writing – original draft, Writing – review & editing.
